# 单操作孔胸腔镜胸腺扩大切除治疗重症肌无力：附45例报告

**DOI:** 10.3779/j.issn.1009-3419.2020.03.04

**Published:** 2020-03-20

**Authors:** 川 黄, 宏峰 佟, 耀光 孙, 青峻 吴, 超 马, 鹏 焦, 文鑫 田, 瀚博 于, 文 黄, 永忠 王

**Affiliations:** 100730 北京，北京医院胸外科，国家老年医学中心，中国医学科学院老年医学研究院 Department of Thoracic Surgery, Beijing Hospital, National Center of Gerontology, Institute of Geriatric Medicine, Chinese Academy of Medical Sciences, Beijing 100730, China

**Keywords:** 重症肌无力, 胸腺扩大切除, 电视胸腔镜, 单操作孔, Myasthenia gravis, Extended thymectomy, Video-assisted thoracoscopic surgery, Single-utility port

## Abstract

**背景与目的:**

胸腺切除已成为重症肌无力（myasthenia gravis, MG）治疗的重要组成部分，近年来，经电视胸腔镜（video-assisted thoracoscopic surgery, VATS）胸腺扩大切除得到广泛应用。传统VATS术式多需3个经肋间切口，本研究改良了手术入路和操作方式，现总结单操作孔VATS胸腺扩大切除治疗MG的效果。

**方法:**

回顾性分析2017年7月-2018年12月北京医院胸外科应用单操作孔VATS行胸腺扩大切除术的45例MG患者资料，总结其手术安全性和疗效。

**结果:**

本组45例均顺利完成胸腺扩大切除，无中转开胸、增加切口和围术期死亡，平均手术时间（141.3±39.2）min，平均术中出血量（64.2±45.5）mL，中位胸腔引流管留置时间3 d，平均胸腔引流量（890.4±439.1）mL，中位术后住院时间6 d。围术期并发症13例（28.9%），其中肌无力危象5例（11.1%），肺部并发症6例（13.3%），切口愈合不良4例（8.9%），房颤4例（8.9%），迟发性心包积血1例（2.2%）。中位随访时间18.5个月，统计术后1年的疗效，药物缓解1例（2.2%），微小症状表现18例（40.0%），改善23例（51.1%），无变化1例（2.2%），加重2例（4.4%）。

**结论:**

单操作孔胸腔镜下胸腺扩大切除术治疗MG的手术安全性和疗效良好，围术期应注意预防肌无力危象、肺部并发症和切口并发症。

## Abstract (en)

**Backgroud and objective:**

Thymectomy plays an important role in patients with myasthenia gravis (MG) and video-assisted thoracoscopic (VATS) extended thymectomy has been widely used in recent years. The traditional thoracoscopic procedure requires three intercostal incisions. Our study improved the surgical approach and operation method by using single-utility port thoracoscopy. The aim of this study was to evaluate the effect of single-utility port VATS extended thymectomy for MG.

**Methods:**

A retrospective analysis was performed in 45 patients with MG who underwent single-utility port VATS extended thymectomy in Beijing Hospital from July 2017 to December 2018. The safety and effectiveness of this surgical approach were summarized.

**Results:**

All operations were accomplished successfully, without conversion to thoracotomy, requirement of additional incisions or postoperative death. The mean operation time was (141.3±39.2) min; the mean intraoperative blood loss was (64.2±45.5) mL; the median thoracic drainage duration was 3 days (range 2 days to 8 days); the mean pleural drainage was (890.4±439.1) mL, and the median postoperative hospital stay was 6 days (range 3 days to 91 days). There were 13 cases (28.9%) with postoperative complications, including 5 cases (11.1%) with myasthenia crisis, 6 cases (13.3%) with pulmonary complications, 4 cases (8.9%) with poor incision healing, 4 cases (8.9%) with atrial fibrillation, and 1 case (2.2%) with delayed pericardial hemorrhage. The median follow-up time was 18.5 months (range 12.5 months to 29.2 months). According to the effect of 1 year after surgery, 1 case (2.2%) showed pharmacologic remission; 18 cases (40.0%) showed minimal manifestations; 23 cases (51.1%) got improvement; 1 case (2.2%) showed no changs and 2 cases (4.4%) were worse.

**Conclusion:**

Single-utility port VATS extended thymectomy is a safe and feasible minimally invasive procedure for MG. During the perioperative period, special attention should be paid to prevent myasthenic crisis, pulmonary complications, and incision complications.

重症肌无力（myasthenia gravis, MG）是由抗体介导的累及神经肌肉接头突触后膜的获得性自身免疫性疾病，胸腺异常在诱导和维持MG发病、发展过程中起重要作用，胸腺切除已成为治疗MG的重要手段之一。传统经胸骨正中劈开入路胸腺切除虽视野直观、疗效确切，但创伤大、恢复慢。近年来，越来越多的研究^[1, 2]^显示经电视胸腔镜（video-assisted thoracoscopic surgery, VATS）胸腺切除与开胸术式疗效相当，得益于其创伤小、恢复快、切口美观、术野清晰等优点，日益广泛应用于胸腺外科领域。MG外科治疗要求完整切除胸腺并彻底清扫周围脂肪，即胸腺扩大切除，传统经VATS术式多需做3个经肋间切口，随着手术技术的成熟，我们改良了手术入路和操作方式，省略腋下切口，仅保留腋前线和锁骨中线的切口，即单操作孔胸腔镜。2017年7月-2018年12月北京医院胸外科完成单操作孔胸腔镜胸腺扩大切除术45例，现将其手术安全性和疗效报道如下。

## 1 资料和方法

### 1.1 入组对象

2017年7月-2018年12月在北京医院胸外科行单操作孔胸腔镜胸腺扩大切除术的MG患者。纳入标准：经我院神经内科行肌无力症状评估、新斯的明试验、肌电图、自身抗体筛查后确诊为MG；术前均行胸部计算机断层扫描（computed tomography, CT）或磁共振成像（magnetic resonance imaging, MRI），可疑合并胸腺瘤者，患者一般情况和心肺功能可耐受手术。排除标准：既往有双侧气胸、胸腔感染、胸部外伤史或手术史，导致双侧胸腔粘连者；肿瘤直径 > 5 cm；肿瘤侵犯重要脏器或出现远处转移；合并其他严重疾病导致手术禁忌者；重要脏器侵犯和远处转移。

手术适应证：MG合并胸腺瘤者宜尽早手术。不合并胸腺瘤者，全身型MG（尤其是乙酰胆碱受体抗体阳性者）可积极选择手术，眼肌型MG经药物治疗无效、有向全身型转化风险者可选择手术。乙酰胆碱受体抗体阴性者，手术疗效不佳，首选药物治疗。术前MG症状严重者（Osserman Ⅱb型-Ⅳ型）经神经内科评估并将MG病情控制稳定后行手术。

### 1.2 入组结果

本组患者45例，男性22例，女性23例，平均体质指数（25.43±4.45）kg/m^2^，中位年龄51岁（15岁-83岁），中位发病年龄51岁（1岁-83岁），中位病程6个月（0.5个月-240个月）。术前合并症17例，其中高血压11例，冠心病3例，糖尿病4例，甲状腺疾病4例，合并肺癌同期手术者1例。改良Osserman分型、胸腺病理、胸腺瘤分型和分期见表 1。

**1:** 45例患者的临床资料 Clinical characteristics of 45 patients

Clinical characteristics	Data
Gender	
Male	22 (48.9%)
Female	23 (51.1%)
BMI (kg/m^2^), Mean±SD (range)	25.43±4.45 (17.15-38.10)
Age of onset (yr), Md (range)	51 (1-83)
Age of operation (yr), Md (range)	51 (15-83)
History of MG (mo), Md (range)	6 (0.5-240)
Osserman classification of MG	
Ⅰ	8 (17.8%)
Ⅱa	13 (28.9%)
Ⅱb	18 (40.0%)
Ⅲ	3 (6.7%)
Ⅳ	3 (6.7%)
Pathological type of thymus	
Hyperplasia	32 (71.1%)
Atrophy	7 (15.6%)
Normal	6 (13.3%)
Thymoma	10 (22.2%)
WHO classification of thymoma	
AB	3 (30.0%)
B1	1 (10.0%)
B2	1 (10.0%)
B2+B3	4 (40.0%)
Micronodular thymoma	1 (10.0%)
Masaoka stage of thymoma	
Ⅰ	4 (40.0%)
Ⅱa	2 (20.0%)
Ⅱb	4 (40.0%)
BMI: body mass index; Md: median; WHO: World Health Organization.	

### 1.3 围术期管理

本组患者围术期均经神经内科治疗，术前使用胆碱酯酶抑制剂40例，溴吡斯的明中位用量180 mg/d（90 mg/d-480 mg/d），糖皮质激素5例，免疫抑制剂（他克莫司、环磷酰胺、硫唑嘌呤）9例。6例Osserman Ⅲ型-Ⅳ型患者术前MG症状严重，经药物控制稳定后再手术，其中使用静脉注射用丙种球蛋白（intravenous immunoglobulin, IVIG）4例，血浆置换1例，糖皮质激素冲击治疗1例。

术毕充分评估患者呼吸肌力恢复程度，呼吸肌力恢复良好者可拔管，呼吸肌力恢复差者则带气管插管至监护病房行呼吸机支持，呼吸肌力恢复后尽早脱机拔管。术后6 h内口服或经胃管鼻饲胆碱酯酶抑制剂，术后使用IVIG 4例，糖皮质激素冲击3例，免疫抑制剂（他克莫司、环磷酰胺、硫唑嘌呤）31例。

### 1.4 手术方法

本组患者采用双腔气管插管或带支气管阻塞球囊的单腔气管插管，静脉吸入复合全身麻醉，手术对侧单肺通气，麻醉诱导时用半量肌松剂，术中无特殊情况则不再追加肌松剂。体位为仰卧位、术侧垫高30°，观察孔为锁骨中线第5或6肋间或肋缘下1 cm切口，操作孔为腋前线第4肋间2 cm-3 cm切口，女性患者注意避开乳腺（图 1）。

**1:**
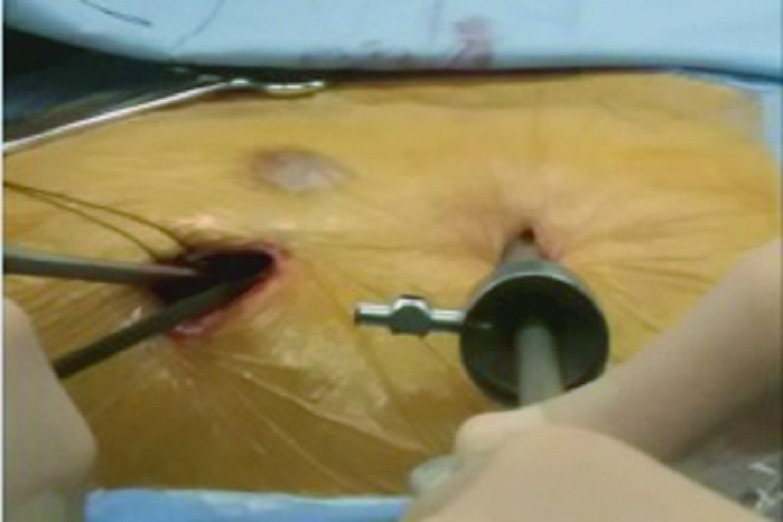
手术体位及切口位置：体位为仰卧位、术侧垫高30°。观察孔为锁骨中线第5或6肋间或肋缘下1 cm切口，操作孔为腋前线第4肋间2 cm-3 cm切口 Surgical position and location of surgical incision: the operation position is supine and the operation side is 30°high. The observation hole is a 1 cm incision in the 5^th^ or 6^th^ intercostal space or under the costal margin of midclavicular line. The operation hole is a 2 cm-3 cm incision in the 4th intercostal space of the anterior axillary line

手术流程（以经右胸VATS为例）：胸腔镜探查后，沿胸骨后缘、右侧膈神经前缘0.5 cm处分别切开右侧纵隔胸膜，沿胸腺右叶向上游离至乳内动脉起始部和无名静脉上缘，完整切除胸腺右叶及右前纵隔脂肪；以同法沿左侧膈神经前0.5 cm处切开纵隔胸膜，完整切除胸腺左叶和左前纵隔脂肪，左侧膈神经显露困难者，可适当向背侧推压心脏，降低左肺潮气量或暂停通气，通常可良好显露；彻底清扫双侧胸腺上极和颈根部脂肪，上缘通常达甲状腺下极，后缘达头臂动脉；彻底清扫前下纵隔脂肪和双侧心膈角脂肪，注意保护膈神经，清扫左侧心膈角脂肪时，可将腔镜置于操作孔，利用观察孔操作器械，可顺利完成操作。

术毕经观察孔留置1根28 Fr引流管于前纵隔，留置1根12 Fr引流管于左后肋膈角，术后第1或2天拍胸片，无气胸、胸腔积液则拔除引流管。

### 1.5 疗效评价和随访

采用美国胸外科医师协会普胸外科数据库的定义^[3]^，统计术后90 d内并发症。术后疼痛评估采用视觉模拟评分（visual analogue score, VAS），定义如下：0分：无痛；3分以下：轻微疼痛，能忍受；4分-6分：疼痛，影响睡眠，尚能忍受；7分-10分：疼痛逐渐强烈，难忍，影响食欲和睡眠。记录第1、2、3天VAS评分和术后止痛药使用情况。术后定期通过门诊或电话随访，疗效评价采用美国重症肌无力协会（Myasthenia Gravis Foundation of America, MGFA）的标准^[4]^。

### 1.6 统计学方法

采用SPSS 22.0软件进行统计分析，正态分布的计量资料以均数±标准差表示，非正态分布的计量资料以中位数表示。

## 2 结果

本组45例均经单操作孔VATS顺利完成胸腺扩大切除术，无增加切口和中转开胸，其中经右胸入路者43例，经左胸入路者2例。平均手术时间（141.3±39.2）min，平均术中出血量（64.2±45.5）mL，无术中输血，中位胸腔引流管留置时间3 d（2 d-8 d），平均胸腔引流量（890.4±439.1）mL，中位术后住院时间6 d（3 d-91 d）。

围术期并发症13例（28.9%, 13/45）。肌无力危象（myasthenic crisis, MC）5例（11.1%, 5/45），术前均为Osserman Ⅱb型-Ⅳ型，经IVIG治疗者2例，糖皮质激素冲击3例，气管插管行机械通气3例，无创呼吸机辅助通气2例，MG病情均明显改善后顺利出院。肺部并发症6例（13.3%, 6/45），其中肺部感染4例，肺不张4例，胸腔积液6例；房颤4例，术后1个月迟发性心包积血1例，返院行心包穿刺引出910 mL积血后缓解；切口愈合不良4例（8.9%, 4/45），包括乙级愈合3例，丙级愈合1例。无术后30 d和术后90 d死亡。术后切口疼痛：术后第1天中位疼痛评分2分（2分-8分），术后第2天中位疼痛评分2分（2分-9分），术后第3天中位疼痛评分2分（2分-7分），术后需使用镇痛药物者12例（26.7%, 12/45）（表 2）。

**2:** 5例患者的围术期结局和术后1年疗效 Perioperative outcomes and postoperative 1-year effects of 45 patients

Perioperative outcomes	Data
Operation time (min), Mean±SD (range)	141.3±39.2 (80-260)
Intraoperative blood loss (mL), Mean±SD (range)	64.2±45.5 (20-200)
Intraoperative blood transfusion	0 (0.0%)
Conversion to thoracotomy or require of additional incisions	0 (0.0%)
Thoracic drainage duration (d), Md (range)	3 (2-8)
Pleural drainage (mL), Mean±SD (range)	890.4±439.1 (250-2, 450)
Postoperative hospital stay (d), Md (range)	6 (3-91)
Postoperative complications	13 (28.9%)
Myasthenia crisis	5 (11.1%)
Pulmonary complications	6 (13.3%)
Atrial fibrillation	4 (8.9%)
Poor incision healing	4 (8.9%)
Delayed pericardial hemorrhage	1 (2.2%)
90-day mortality	0 (0.0%)
Postoperative VAS pain score, Md (range)	
Postoperative day 1	2 (2-8)
Postoperative day 2	2 (2-9)
Postoperative day 3	2 (2-7)
Postoperative analgesia	12 (26.7%)
Postoperative 1-year effects	
Pharmacologic remission	1 (2.2%)
Minimal manifestations	18 (40.0%)
Improved	23 (51.1%)
Unchanged	1 (2.2%)
Worse	2 (4.4%)
VAS: visual analogue score.	

MG疗效：末次随访日期2019年12月20日，中位随访时间18.5个月（12.5个月-29.2个月）。统计术后1年的疗效，药物缓解1例（2.2%），微小症状表现18例（40.0%），改善23例（51.1%），无变化1例（2.2%），加重2例（4.4%）（表 2）。

## 3 讨论

胸腺在诱导和维持MG发病、发展过程中起重要作用，约80%-90%的MG合并胸腺异常，胸腺切除（thymectomy, Tx）已成为MG治疗的重要组成部分。2016年发表的全球多中心、随机对照的MGTX研究^[5]^显示，与单纯药物治疗相比，手术不仅可改善术后3年平均定量MG评分，还明显降低糖皮质激素用量、免疫抑制剂使用率和MG病情加重导致的再住院率，其结果强有力地支持了Tx在MG治疗中的地位。本研究涵盖Ⅰ型-Ⅳ型MG，术后1年有效率（药物缓解+微小症状表现+改善）达93.3%，其中53.3%（24/45）的患者为病情较重的IIb型以上MG，经内外科合作完成围术期管理，均获得良好疗效。

长久以来，经胸骨正中劈开入路Tx是治疗MG的标准术式，但该术式创伤大、恢复慢、疼痛明显、美观性差。1995年Yim等^[6]^首次尝试了经胸腔镜行Tx治疗MG，开创了胸腺外科的微创时代。大宗报道^[1, 2]^显示微创Tx可减少手术出血量、缩短胸管留置时间和住院时间，在围术期并发症、MG疾病缓解率、胸腺瘤R0切除率、复发率及5年生存率等方面达到与开胸术式相同的疗效。因此，微创Tx以其创伤小、恢复快、术野清晰、切口美观等优点，日益广泛应用于胸腺外科领域。我们首选经右胸入路VATS手术，对纵隔大血管、膈神经等重要组织显露良好，手术时间短，失血量少，无严重并发症，手术安全可行；88.9%（40/45）的患者在术后4 d内拔除引流管，77.8%（35/45）的患者在术后7 d内出院，恢复速度快，尤其适用于MG病情重、肺功能差、高龄的患者。

经肋间入路的微创Tx术式多采用三孔法，即在腋下、腋前线和锁骨中线处做3个切口，切口分布于2个-3个肋间，尤其腋下切口面临肌层厚、易出血、肋间狭窄、易损伤肋间血管神经、增加术后疼痛及不适感等问题。我们改良了切口设计，取消腋下操作孔，以腋前线切口作为操作孔，锁骨中线切口为观察孔，尽量采用带弧度、双关节、细杆腔镜器械，灵活调换器械和腔镜位置，从而减少器械间干扰，显露良好，操作便利。本组仍有26.7%（12/45）的患者术后需使用镇痛药物，其中4例仅需口服非甾体类镇痛药，另8例则需盐酸哌替啶、氨酚羟考酮等强效镇痛药，使用不经肋间的剑突肋缘下切口有助于进一步减轻疼痛，国内唐都医院报道^[7, 8]^经剑突肋缘下入路Tx可降低切口疼痛和切口感染，显露良好，操作便利，美容性好。本组4例患者术后切口愈合不良，可能与其中2例术前长期使用糖皮质激素、1例术前长期使用免疫抑制剂有关。

MC是围术期主要并发症之一，文献[9, 10]报道胸腺切除术后MC发生率为10%左右，本组11.1%（5/45）的患者术后出现MC，该5例患者术前MG症状重，均已累及延髓肌或呼吸肌，其中Osserman Ⅲ型1例、Ⅳ型1例术前经IVIG治疗，另有Osserman Ⅱb型3例术前经溴吡斯的明和免疫抑制剂治疗，术前有效控制MG症状有助于降低术后MC发生率。对于高危病例，术后需谨慎把握气管插管拔除时机，拔管前后均应密切关注全身肌力，加强气道管理，避免肺部并发症，MC起病隐匿，早期常表现为高碳酸血症，定时监测动脉血气有助于早期诊断。一旦出现MC，宜早期干预，积极行无创或有创呼吸机支持，综合使用IVIG、糖皮质激素冲击或免疫抑制剂，并给予全身支持治疗。本组5例MC患者均顺利出院，术后1年评效均较术前明显改善（微小症状表现2例、改善3例），远期疗效良好。

Tx清扫不彻底导致的胸腺残留是术后病情持续或复发的重要根源，因此MG手术关键和难点在于彻底清除胸腺及胸腺周围脂肪，避免异位胸腺残留。与开胸术式相比，单侧入路VATS手术在显露对侧膈神经和心膈角脂肪时存在困难，增加膈神经损伤几率和心膈角脂肪清扫难度，有研究^[11, 12]^报道双侧入路VATS手术清扫纵隔脂肪更彻底，有助于提高MG缓解率，但增加手术创伤，延长手术时间。我们首选经右胸入路VATS，避免经左胸入路时心脏大血管的干扰，上腔静脉及双侧无名静脉显露清楚，可彻底清扫胸腺上极及颈根部脂肪；靠近左侧膈神经操作时可适当向背侧推压心脏、降低或暂停左肺通气，通常可良好显露，推荐使用电钩而非超声刀，靠近膈神经操作时即可刺激膈肌活动从而避免损伤神经；清扫左侧心膈角脂肪时，可将腔镜移至腋前线切口，术者经腋前线操作孔和锁骨中线观察孔进入器械，适当推压心包和膈肌即可清晰显露、顺利完成清扫。本组有2例胸腺瘤患者因肿瘤位置偏左、邻近左侧膈神经，为便于显露而采用经左胸入路，切口设计时应注意将操作孔和观察孔尽量远离心脏大血管，从而减少心脏搏动和大血管干扰，保持观察视野和操作稳定。本组采用上述方法，均顺利完成手术，无纵隔大血管出血、膈神经损伤等并发症，手术安全、有效。但本组有1例患者术后1个月出现心包积血，可疑为紧邻心包游离时损伤心包内血管导致，提示沿心包表面操作不宜过快，应精准止血，避免血管断端迟发出血，术后出现心悸、憋气等不适需及时检查，一旦确诊，应早期行心包引流或手术探查。

值得注意的是，单操作孔VATS术式不适用于既往合并胸腔疾病史、手术史导致胸腔粘连者；体质指数大、纵隔脂肪多的患者，对侧膈神经显露欠佳，增加损伤风险；严重冠心病、冠脉钙化严重、大血管多发斑块的患者，操作时应尽量减少对心脏和纵隔大血管过度挤压，避免血管斑块脱落、栓塞等严重并发症。肺部并发症和MC仍是MG外科围术期的主要并发症，尤其是病情严重的IIb型以上MG患者，术前需有效控制MG病情，术后应重视呼吸道管理，长期使用激素和免疫抑制剂者应格外关注切口和感染性并发症。本研究是单中心、小样本的回顾性研究，存在偏倚等不足，仍需大样本、前瞻性的随机对照研究验证。

综上所述，单操作孔VATS可顺利完成胸腺及周围脂肪的扩大清扫，术野显露良好、操作便利、安全可行，围术期应关注肌无力危象、肺部并发症和切口并发症，单操作孔VATS治疗MG的远期疗效良好。

## Author contributions

Huang C and Tong HF conceived and designed the study. Huang C performed the experiments. Huang C analyzed the data. Huang C contributed analysis tools. Wu QJ, Jiao P, Ma C, Sun YG and Tong HF provided critical inputs on design, analysis, and interpretation of the study. All the authors had access to the data. All authors read and approved the final manuscript as submitted.
